# Gut–Skin Axis: Unravelling the Connection between the Gut Microbiome and Psoriasis

**DOI:** 10.3390/biomedicines10051037

**Published:** 2022-04-30

**Authors:** Angel Yun-Kuan Thye, Yi-Rou Bah, Jodi Woan-Fei Law, Loh Teng-Hern Tan, Ya-Wen He, Sunny-Hei Wong, Sivakumar Thurairajasingam, Kok-Gan Chan, Learn-Han Lee, Vengadesh Letchumanan

**Affiliations:** 1 Novel Bacteria and Drug Discovery Research Group (NBDD), Microbiome and Bioresource Research Strength (MBRS), Jeffrey Cheah School of Medicine and Health Sciences, Monash University Malaysia, Bandar Sunway, Selangor Darul Ehsan 47500, Malaysia; angelthye.yunkuan@monash.edu (A.Y.-K.T.); bah.yirou@monash.edu (Y.-R.B.); jodi.law1@monash.edu (J.W.-F.L.); loh.teng.hern@monash.edu (L.T.-H.T.); vengadesh.letchumanan1@monash.edu (V.L.); 2 Clinical School Johor Bahru, Jeffrey Cheah School of Medicine and Health Sciences, Monash University Malaysia, Johor Bahru 80100, Malaysia; sivakumar.thurairajasingam@monash.edu; 3 State Key Laboratory of Microbial Metabolism, Joint International Research Laboratory of Metabolic and Developmental Sciences, School of Life Sciences & Biotechnology, Shanghai Jiao Tong University, Shanghai 200240, China; yawenhe@sjtu.edu.cn; 4 Lee Kong Chian School of Medicine, Nanyang Technological University, Singapore 308232, Singapore; sunny.wong@ntu.edu.sg; 5 State Key Laboratory of Digestive Disease, Department of Medicine and Therapeutics, The Chinese University of Hong Kong, Hong Kong SAR, China; 6 Division of Genetics and Molecular Biology, Institute of Biological Sciences, Faculty of Science, University of Malaya, Kuala Lumpur 50603, Malaysia; 7 International Genome Centre, Jiangsu University, Zhenjiang 212013, China

**Keywords:** microbiota, skin disease, autoimmune disease, probiotic, gut dysbiosis

## Abstract

Evidence has shown that gut microbiome plays a role in modulating the development of diseases beyond the gastrointestinal tract, including skin disorders such as psoriasis. The gut–skin axis refers to the bidirectional relationship between the gut microbiome and skin health. This is regulated through several mechanisms such as inflammatory mediators and the immune system. Dysregulation of microbiota has been seen in numerous inflammatory skin conditions such as atopic dermatitis, rosacea, and psoriasis. Understanding how gut microbiome are involved in regulating skin health may lead to development of novel therapies for these skin disorders through microbiome modulation, in particularly psoriasis. In this review, we will compare the microbiota between psoriasis patients and healthy control, explain the concept of gut–skin axis and the effects of gut dysbiosis on skin physiology. We will also review the current evidence on modulating gut microbiome using probiotics in psoriasis.

## 1. Introduction

Psoriasis is a non-contagious chronic inflammatory skin condition with a complex etiology that is relatively common in the general population, affecting men and women of all ages, regardless of ethnic origin, in all countries [1,2]. Psoriasis is a T cell mediated disease involving Th17 cells secreting interleukin (IL)-17A and IL-22 which are proinflammatory cytokines that causes proliferation of keratinocyte (KC) and activation of synoviocyte [3,4]. Psoriasis can be characterized by the hyperproliferation of the epidermal KCs, dysregulated KC differentiation, elevated vascularization and inflammation of the dermis and epidermis resulting in thickened, reddened skin appearing as a classic, well defined, erythematous scaly plaque that is itchy and flaky [5,6,7]. Although clinical findings are noticeable on the outer layer of the skin that is composed of KCs, the formation of psoriatic plaque is an interplay between different cell types (vasculature, innate, and adaptive immune cells) and KCs across the dermal layer rather than just the epidermal inflammation [8]. Risk factors of psoriasis include family history [9], smoking [10], obesity [11,12], infections [13,14], and medications [13]. According to the World Health Organization (WHO), nearly 100 million individuals globally are affected by psoriasis [1]. The reported prevalence in countries ranges between 0.09% and 11.4% [1,15]. Some studies find the prevalence rate of psoriasis could be affected by regions as Asians and some African populations showed lower prevalence rate as compared to Scandinavian and Caucasians that have prevalence rates as high as 11% [15,16,17]. However, one study found the correlation between psoriasis prevalence and geographic latitude to be very weak [18]. In terms of the mean age of onset of psoriasis, although there are variations across different studies, 75% of patients were <40 years old and 12% were between 50–60 years old [16].

The concept of gut–skin axis which relates gut microbiome with skin health has garnered remarkable interest amongst researchers. The association between inflammatory skin diseases and gut microbiome is known to be mediated by dysfunctional intestinal barrier, increased inflammatory mediators and metabolites released by the microorganisms [19,20,21]. The interplay between gut microbiota and immune system has been well established. Gut microbiome plays an important role in the immune system development and regulation of immune homeostasis through its interaction with the innate and adaptive components of the immune system [22]. Disturbance to the gut microbiome or changes to the host–microbiome interfaces may trigger an immune response and increase risk of pathogenic invasion [22,23]. Both systemic and local inflammation can be caused by alterations of the microbiota on the epithelial surface, leading to systemic disease susceptibility [19]. For instance, in patients with Inflammatory Bowel Disease, local inflammation caused by the increased pro-inflammatory bacteria at gut epithelium leads to mucosal damage and increased permeability of gut mucosa [24]. The damage of gut mucosal layer subsequently causes a surge in pro-inflammatory cytokines such as IL-12 and IFN-γ, leading to systemic inflammation [24]. Although psoriasis is a disorder of the skin, it is recognized as a systemic inflammatory disease [25], as it results in the inflammation of other organ systems in addition to psoriatic skin. Furthermore, psoriasis has been associated to several metabolic disorders [7,16,17]. This is seen as psoriasis patients show greater body mass index, hypertension, hyperlipidemia, type 2 diabetes, and coronary artery disease [26,27]. All of these effects together with obesity and inflammatory bowel disorders are psoriasis comorbidities [7,28,29,30,31,32]. Currently, psoriasis is a disorder with no curative treatment and it can only be suppressed using various therapeutics [33], thus, it definitely impacts the quality of life of psoriasis patients’ physically and psychologically.

In recent years, many studies have been investigating the connection between skin allostasis and homeostasis and the gastrointestinal health, with corroborative evidence showing strong bidirectional relationship between the skin and gut [34,35]. This can be seen through the presence of bacterial DNA translocation (BT) in blood samples of psoriasis patients that has been associated with the composition of gut microbiome in recent years, which proposes the new outbreaks of active plaque psoriasis could be correlated to circulating bacterial DNA in blood from the intestinal lumen [36]. The advancement in next generation sequencing technologies in the past few years has allowed us to have a better understanding on the intestinal microbiota composition [37], and the effects of these microbes may have on psoriasis pathogenesis. Hence, this review discusses the gut microbiome composition, diversity, and relative abundance of healthy and psoriasis individuals, explains the gut–skin axis and the effects gut dysbiosis has on the epithelial barrier, gut microbial metabolite and the gut immunoregulatory characteristics. We will also discuss the benefits of modulating gut microbiome using probiotics and how it can improve symptoms of psoriasis.

## 2. Cutaneous Immune System and Psoriasis Pathogenesis

The skin is one of the largest organs of the human body. The skin plays a vital role in homeostasis in terms of water retention, temperature regulation, and protection of the body via skin regeneration process [38,39]. It also helps maintain a healthy microbial ecosystem via its production of antimicrobial proteins and peptides [40]. The role of homeostasis is highly dependent on the stratum corneum—the outermost layer of the epidermis—which is made up of 15 layers of tightly packed keratinized, anucleated, and stratified corneocytes differentiated from stem cells in the basal layer through the keratinization process [38,39,41,42].

The skin immune system is made up of recruited and resident innate immune system (IIS) and adaptive immune system (AIS) cells which are activated by microorganisms, stimuli, and epidermal structures that crosstalk with mostly KCs to restore skin barrier [43,44]. IIs releases signals initiating skin immune response while AIS activation prolongs inflammation [45]. There are postulations that psoriasis has mixed pathogenesis of autoinflammatory and autoimmune states [46]. The pathogenesis of psoriasis probably involves cross-talking of the skin’s complex network of dendritic cells (DCs), resident KCs and T cells (mostly Th17 [47]), that gives rise to immune and inflammatory route accountable for the initiation, progression and persistence of psoriasis [48,49,50]. This development of inflammation occurs due to interference in the innate and adaptive cutaneous immune responses [8,51].

Janus kinases (JAKs) signaling, nuclear factor kappa (NF-κB) signaling, transforming growth factor beta (TGF-β), interleukin 23–interleukin 17 (IL-23-IL-17) signaling, T cell regulation, disruption of epithelial barrier function, autophagy, and dysregulated apoptosis are all involved in psoriasis pathogenesis [52,53,54]. Exposure of self-nucleic acids to tissue occurs as epithelial cells undergo necrosis or apoptosis after exposure to virus, bacteria, mechanical stress, or ultraviolet light. Self-DNA bound to LL-37 which is produced by KCs and is a part of the antimicrobial peptide cathelicidin that stimulates production of type 1 interferons by plasmacytoid dendritic cells (pDC). Concurrently, self-RNA bound to LL-37 stimulates myeloid dendritic cells (mDC), producing inducible nitric oxide synthase (iNOS) and tumor necrosis factor (TNFα). The production of these cytokines leads to immature T cells to transform into inflammatory T cells (mostly Th17) producing IL17 and IL-22, developing psoriatic phenotype in KCs. KCs produces proinflammatory cytokines (1L-1, IL-17 and TNFα), chemokines (CXCL20,11,10,8,2, and CXCL 1) and antimicrobial peptides (S100 proteins, psoriasin, cathelicidine, and beta defensin (BD) that draws in Th17 cells and neutrophils resulting in sustained chronic psoriasis [55].

In lesional psoriatic skin, molecular and critical cellular pathways are brought about by the activation of dermal dendritic cells secreting IL-23 to stimulate type 3 innate lymphoid cells (ILC3) and gamma delta T cells to produce IL-17 which cause production of chemokines- interleukin 6 (IL-6), interleukin 8 (IL-8), CXCL20, CXCL2, and CXCL1- by keratinocyte, leading to leukocyte infiltration. With the presence of stimulating cytokines IL-18, IL23 and IL-1β, ILC3 releases IL-17 and IL-22 promoting keratinocyte hyperproliferation [56]. Thus, in comparison to healthy normal skin that takes about 50 days for the transformation of basal KCs to corneocytes, psoriatic skin takes only 5 days [57].

There is a possibility that the modulatory effects of skin flora on inflammatory skin diseases may be associated with the gut microbiota. Imbalances of the composition of skin microbiota has been observed in numerous non-infective skin conditions such as psoriasis, acne vulgaris and rosacea, where gut dysbiosis were too, apparent in these conditions [34]. However, there is currently no evidence to show the direct causality of the association between gut dysbiosis and skin dysbiosis. In the following sections, we will discuss the possible mechanisms on how gut resident commensals affect skin health.

## 3. The Gut Microbiome and the Gut–Skin Axis

In recent years, an increasing number of studies are actively investigating the relationship between the gut microbiome and skin diseases, including psoriasis. This leads us to the concept of gut–skin axis which associates the microbiome and skin diseases via intestinal barrier, inflammatory mediators, and metabolites [21]. Currently there has been large evidence regarding the presence of the gut–skin axis and its resulting inflammatory effect due to gut microbiome imbalance [20]. The gut microbiome is mainly made up of a diverse bacterial species, but also contains protozoa, viruses, and fungi that reside mainly in the lower gut and help to maintain a symbiotic relationship with the host [58,59,60]. Aerobic species are commonly found in the small intestine whereas anaerobic species are common in the colon [61]. The few main bacterial communities in the gastrointestinal tract (GIT) include Firmicutes, Bacteroidota (formerly known as Bacteroidetes), Actinobacteria, and Proteobacteria phyla in which their composition is influenced by the host’s diet, age, and environmental conditions [62,63,64,65].

Dietary, lifestyle, and genetic predisposition are key regulators of gut microbiome homeostasis [66,67]. It has been proven that the gut microbiome is essential in regulating the intestinal permeability, metabolism, and immune system [68,69,70,71]. The gut microbiome ensures the protection against potential pathogens indirectly by triggering immuno-protective responses and directly by binding competitively to epithelial cells and allow for immune tolerance of environmental and dietary antigens [72,73,74,75]. An imbalance of composition and biodiversity of the gut microbes or the term “gut dysbiosis” has been associated with psoriasis and many other psoriasis-associated comorbidities such as inflammatory arthritis, chronic kidney disease, inflammatory bowel disease, metabolic syndrome, cardiovascular disease, depression, and obesity [24,33,76,77,78,79,80].

The gut microbiome could affect skin homeostasis through systemic immunity modulation [34]. Numerous gastrointestinal diseases have been accompanied by cutaneous manifestations and the gut microbiome’s interaction with the immune system, impacting the pathophysiology of inflammatory diseases [81,82,83]. Gut dysbiosis causes negative impacts on the skin integrity and function [84,85]. Some microbes affect the intestinal barrier function and skin homeostasis via cross-talking with mucosal immunity elements and signaling pathways coordinating epidermal differentiation [34,86,87,88,89]. Besides that, there are studies that disseminate the gut microbes and their metabolites onto the skin to demonstrate their effects on the cutaneous physiology, immune system and pathology [34,90]. For instance, metabolites such as p-cresol and phenol produced by *Clostridioides difficile* (formerly known as *Clostridium difficile*) are biomarkers of gut dysbiosis has been shown to enter the bloodstream and accumulate on the skin, decreasing skin moisture, impairing skin barrier integrity and epidermal differentiation and affecting keratinization [91,92]. Hence, it is certain that the gut microbiome is associated with the skin homeostasis and does affect distant organs beyond the GIT.

The relationship between the gut microbiome and the pathogenesis of psoriasis is based upon the association between components of the innate and adaptive immune systems [68,69,70,73,77,78,79]. Studies have proposed that the mechanism of the gut–skin axis in regards to psoriasis involves T cells function and differentiation with the imbalance of Treg and Th17 cells [93,94]. Interaction between pattern recognition receptors expressed by host cell and bacterial antigen enables the gut immune system to be primed by commensal bacteria [73]. The adaptive immunity is affected as these commensal bacteria ensure the balance of effector T cells and regulatory T cells and immunoglobulin A induction leading to B cells activation and thus specific immunoglobulin A antibodies production [73,95]. An experimental model has also demonstrated that gut dysbiosis aids in Th17-mediated skin inflammation [93,94], as well as affecting metabolite production, inducing an anti-microbial signaling changing immune cell activation through IL-23/IL-17 signaling pathway through IL-22 and interferon gamma (IFN-γ) production, resulting in hyperproliferation of keratinocytes [66,67].

In addition, there are a number of studies regarding the concept of gut–skin axis which showed that gut dysbiosis can induce inflammatory skin diseases [20]. One of the many mechanisms by which gut microbiome may cause skin impairment is presented in animal studies with evidence demonstrating that gut dysbiosis causes chronic systemic inflammation as a result of pro-inflammatory cytokine secretion causing an imbalance between activated effector T cells and increased epithelial permeability [34,73]. Intestinal barrier dysfunction and subclinical gut inflammation can be observed in psoriasis patients, and thus, this lead to the postulation that gut dysbiosis is associated with psoriasis [96,97].

Gut dysbiosis activates the proinflammatory state via alterations to the metabolic environment and activation of specific pattern recognition receptors (PRPs) present on epithelial cells. This causes the gut permeability to increase as cytokines such as TNF alter the integrity of tight junctions between epithelial cells. The increase in epithelial permeability stimulates effector T cells activation, causing an imbalance between the T cells and Treg cells which leads to autoimmune diseases development. A positive feedback mechanism is involved as the proinflammatory cytokines magnifies the epithelial permeability, which further exacerbate chronic systemic inflammation and thus greater impairment to the intestinal barrier resulting in the entry of metabolites, toxins, and bacteria into the systemic circulation [34,73,98]. As these microorganisms enter the circulation, they could be activated, shedding their inflammatory cell wall components (lipoteichoic acid and lipopolysaccharide), possibly promoting or maintaining the pro-inflammatory state [99]. On top of that, gut dysbiosis can produce endotoxin-peptidoglycan superantigens to stimulate inflammatory and autoimmune states related to psoriasis. The microorganisms in the gut produce toxins triggering an immune response that causes psoriatic patients to present with positive detection of gut bacterial antigen in a skin test [100]. In line with this model, biomarkers for intestinal permeability such as claudin 3 and fatty acid binding protein are elevated in psoriasis patients [99].

### Effects of Gut Dysbiosis on Gut Microbial Metabolite and the Gut Immunoregulatory Characteristic

The gut microbiome plays a role in the immunoregulatory characteristics of the gut. Gut microbes may produce or even increase the beneficial metabolites or specific immune modulating molecules such as polysaccharide A, short chain fatty acids (SCFAs) and retinoic acid via the fermentation of dietary fibers [73,101,102,103]. They are involved in the homeostasis between effector and regulatory T cells [73,102], aiding the anti-inflammatory responses via upregulation of lymphocytes and regulatory T cells [101]. However, the specific microbes involved in the modulation of these immune modulating molecules for such mechanism seen in psoriasis are yet to be distinguished [19]. The production of short chain fatty acids (SCFAs) and trimethylamine can affect disease state and health status of a subject [76]. SCFAs have a role in protecting against the progression of certain inflammatory disease [103]. For example, propionate and butyrate produced by the gut microbiota have been shown to have anti-inflammatory properties [104]. Butyrate has the key role of maintaining barrier integrity [105], as it can cease the activity of histone deacetylase causing a rise in regulatory cells which impacts wound healing and hair follicle stem cell differentiation [100]. Butyrate, which is also known to be primarily produced by *Faecalibacterium prausnitzii*, functions to decrease oxidative stress, supplies energy for colonocytes and triggers Treg cells, allowing anti-inflammatory action, hence, conferring immune tolerance to sites other than the GI system [106,107]. Consequently, a drop in both propionate and butyrate microbiota producers can trigger a proinflammatory state of the gut and affect the gut barrier integrity [108]. In addition, SCFAs are involved in the apoptosis and activation of immune cells. Evidence of chronic systemic inflammation demonstrated in animals is the main consequence of intestinal dysbiosis, due to the secretion of pro-inflammatory cytokines causing epithelial to be more permeable and effector T cells to be activated [34,73]. By taking sodium butyrate as an example, it has important effects on tumor growth factors (TGF-β), protease enzymes, and cell cycle. Several studies showed that by exposing sodium butyrate to human keratinocyte (HaCaT) cells, it prompts apoptosis by 50% via death receptors Fas upregulation accompanied with activation of caspases 3 and 8. It also helps in cell proliferation and terminal differentiation as demonstrated in the rise in expression levels of TGF-β and p52 [109].

## 4. Alterations in Alpha and Beta Diversity of Gut Microbiome in Psoriasis Patients

In many microbiome-profiling studies, the diversity indices allow further characterization of microbiota population [110]. In terms of assessing the alpha diversity using Shannon’s Diversity index of the gut microbiome in psoriasis, a systematic review reported that in 8 out of 10 studies that looked at alpha diversity, most of them failed to demonstrate remarkable changes between psoriasis and normal control [21,102,111,112,113]. However, only one study among them showed increased diversity [108], two other studies showed lower diversity [114,115], and one study presented similar diversity but lower community richness in psoriatic samples when compared with normal controls [116]. There was also high variability in terms of Shannon’s biodiversity index of the psoriatic patients, in which bacterial DNA translocation positive psoriatic patients having a more stable and lower variability in diversity as compared to BT negative psoriatic patients. According to Codoner et al., in a human microbiome project of 300 healthy controls (HC) and 52 psoriasis subjects, the microbiome diversity of psoriasis patients was found to be greater than the healthy controls [108]. However, according to Scher et al. whose study involved only 17 HC, 15 psoriasis subjects, and 16 psoriatic arthritis subjects, results show psoriasis subjects had lower microbial diversity [114]. This is consistent with study conducted by Hidalgo-Cantabrana et al. which found that psoriasis patients presented with severe dysbiosis, a lower diversity of gut microbiota and an alteration of the relative abundance for some bacterial taxa [115]. Therefore, even with a similar alpha diversity index, microbial communities can still have a shift in composition without sharing any taxa [117]. To sum it up, there were no significant differences in alpha diversity between healthy controls and psoriasis individuals based on similar indices of most studies [21]. However, there are conflicting data regarding the alpha diversity which could be due to differences in sequence library preparation, DNA extraction, sample collection and data analyses [40]. Besides that, it is hypothesized that instead of the number of bacterial species, the differential abundance of bacteria may be the cause of gut dysbiosis in psoriasis [21].

On the other hand, beta diversity differed significantly between psoriasis and healthy controls in all studies included in the systematic review [21]. Having said that, it was reported that the differences in beta diversity achieve statistical significance only for psoriatic patients who have BMI < 25 [21].

## 5. Alterations in Relative Abundance of Gut Microbiome of Psoriasis Patients

In a study conducted by Codoner et al. which involves analyzing the feces of 52 psoriatic patients via 16S rRNA, an average of 85,000 sequences per sample was found and the “psoriasis microbiome” which is the defined microbial structure of psoriatic patients were different compared to healthy individuals. Hence, differing in gut microbial composition, which was reported to also be linked with BT [108].

A number of studies concluded revealed that there is a relationship between gut dysbiosis and psoriasis [21]. There are studies demonstrating an inverse relationship in the relative abundance of Bacteroidota and Firmicutes at the phylum level as well as the presence of 16 phylotypes differing at the genus level [116]. It was found that at the phylum level, the relative abundance of Bacteroidota were lower and the relative abundance of Firmicutes were higher in psoriasis patients [102,112,115]. However, study by Huang et al. states vice versa [116]. This could be due to the small and diverse sample size that includes other psoriasis variants such as pustular, arthritis, plaque, and erythrodermic [21]. When it comes to Proteobacteria, the level was decreased in psoriatic patients [102,115]. Actinobacteria on the other hand had conflicting results in which some had an increased level [102,115], and some had a drop [114,118]. The drop in Actinobacteria presented by some studies [114,118], proposes that Actinobacteria has a protective role as it includes *Bifidobacterium* spp. that could suppress autoimmunity, decrease intestinal inflammation and induce Tregs [119,120].

At the family level, the relative abundance of some gut bacteria increased, for example, *Enterococcaceae* [111], *Ruminococcaceae*, *Lachnospiraceae* [112,115], *Coriobacteriaceae*, *Eggerthellaceae*, *Peptostreptococcaceae*, and *Clostridiales Family XIII* [115], whereas others, such as *Prevotellaceae* [112,115], *Lactobacillaceae*, *Desulfovibrionaceae*, *Pasteurellaceae*, *Barnesiellaceae*, *Rikenellaceae*, *Marinifilaceae*, *Burkholderiaceae*, *Victivallaceae*, *Tannerellaceae*, *Streptococcaceae* [115], *S24-7*, *Verrucomicrobiaceae* [111], and *Porphyromonadaceae* [114], decreased [21]. There were conflicting reports on the changes of relative abundance of the following bacteria families, namely *Bacteroidaceae*, *Veillonellaceae*, *Erysipelotrichaceae,* and *Bifidobacteriaceae.* Some studies reported that these families were increased in patients with psoriasis [111,115], while some reported reduction of these families in psoriasis [112,114,115].

**Figure 1:**
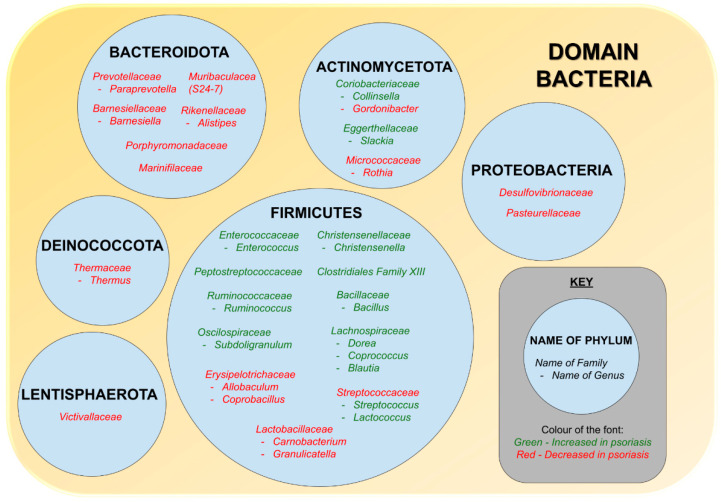
The association between gut microbiota and psoriasis. Data extracted from Scepanovic et al. [110], Calcinaro et al. [119], Lavasani et al. [120], Kostic et al. [122], Scher et al. [123], and Eppinga et al. [124].

At the genus level, some of the bacteria with increased relative abundance are *Bacillus*, [116] *Subdoligranulum*, *Slackia* [115], *Christensenella*, *Dorea*, *Coprococcus* [102], *Collinsella*, *Blautia*, *Ruminococcus* [102,115], *Streptococcus* [116], *Enterococcus* [111], and *Lactococcus* [116], whereas those whose relative abundance dropped are *Allobaculum*, *Alistipes*, *Barnesiella* [115], *Gordonibacter*, *Carnobacterium*, *Rothia*, *Thermus*, *Granulicatella* [116], *Coprobacillus* [114], and *Paraprevotella* [102,115]. However, there were conflicting findings [21], for *Parabacteroides* [114,115,116], *Lachnospira*, *Sutterella* [102,116], *Bacteroides* [108,111,115], *Faecalibacterium* [102,108,115], *Akkermansia* [108,111], and *Bifidobacterium* [102,114,115]. Studies by Codoner et al. showed a decrease in *Bacteroides* but an increase in *Faecalibacterium*, *Ruminococcus,* and *Akkermansia* in psoriatic patients via PCR analysis [108]. However, Scher et al. found *Pseudobutyrivibrio*, *Ruminococcus,* and *Akkermansia* to be lower in both psoriasis patients and psoriatic arthritis patients [114]. Although the gut microbiome composition of skin limited disease (i.e., psoriasis) is different from those of psoriatic arthritis [114], these changes in the gut microbiome are in fact similar to IBD which is one of psoriasis’s comorbidities [114,121]. Both *Ruminococcus* and *Akkermansia* are mucin-degrading bacteria producing SCFA’s that are essential in maintaining the gut mucosal barrier [114,122]. Besides that, Scher et al. also found *Akkermansia* to have an inverse relationship with SCFAs (butyrate, acetate) and fecal soluble IgA [114]. The association between gut microbiota and psoriasis based on phylum, genus, and family level is illustrated in Figure 1.

**Figure 2:**
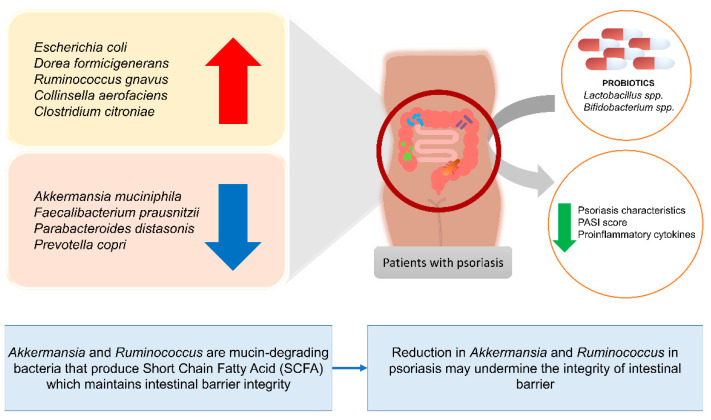
Changes in the abundance of specific bacteria species in patients with psoriasis and the effects of certain probiotic strains on patients with psoriasis.

Lastly, at the species level, those found to drop significantly in psoriasis patients are *Akkermansia muciniphila* [111], *Faecalibacterium prausnitzii* [123,124], *Parabacteroides distasonis*, and *Prevotella copri* [102], while *Escherichia coli* [124], *Dorea formicigenerans*, *Ruminococcus gnavus*, *Collinsella aerofaciens* [102], and *Clostridium citroniae* [111], were increased [21], as shown in Figure 2. In a study analyzing the microbial composition of healthy controls and vulgaris psoriasis patients conducted by Tan et al., it was found that psoriasis patients had a tremendous drop in *Akkermansia muciniphila* [111]. The drop in *Faecalibacterium prausnitzii* was consistent in two studies [123,124]. Psoriasis is affected by the drop in *Akkermansia muciniphila* and *Faecalibacterium prausnitzii* as these bacteria are considered a beneficial microbe responsible for SCFA production thus are protective against systemic inflammatory diseases including IBD, atherosclerosis, and obesity, and is vital in strengthening gut epithelium integrity [107,111,125,126,127,128,129,130].

In addition to bacteria, virus including the human papilloma virus and fungus for example *Candida albicans* and *Malassezia* have been connected to psoriasis [13]. The rise in these fungus and *Staphylococcus aureus* in both gut and skin has been linked with psoriasis exacerbations [13].

## 6. Treatment

There are several treatments for psoriasis and antiproliferative is one of the focused methods in the past as it was thought that psoriasis occurred solely due to skin hyperproliferation. However, as more studies were conducted, the treatment for psoriasis shifted to targeting Th17 cells as there were higher levels of IL-17 found in psoriatic lesions. Th17 releases cytokines that stimulate IL10, IL20, and IL22 cytokines expression which results in keratinocyte hyperproliferation. This leads to more studies showing evidence that psoriasis is mainly driven by IL17/IL23/Th17 axis [131,132,133,134,135]. Hence, biologics targeting key cytokines, for instance, tumor necrosis factor alpha (TNF-α), interleukin-17 (IL-17), and interleukin-23 (IL-23), is a treatment option for psoriasis [136,137,138,139]. Antibiotics, on the other hand, decrease susceptible bacterial species by changing the composition of microbiome, as well as potentially decreasing cardiometabolic comorbidities risk of psoriasis patients [140]. For instance, a study found that after antibiotics administration, TMAO drops then bounce back to baseline when antibiotics are ceased [141].

### Benefits of Gut Microbiome Modulation on Skin Health

With the current evidence suggesting that systemic diseases can be modulated by altering the cutaneous and gut microbiome [19], further understanding of the role of gut microbiome in psoriasis could possibly lead to new therapies to be discovered. Currently, probiotics have been demonstrated to improve psoriasis by alteration of gut microbiota. The utilization of probiotics in psoriasis had shown to provide improvements but treatment using probiotics has yet to be standardized due to variations in studies in terms of the content of probiotic supplements and methods used [20,142,143]. By modifying the gut microbiome using dietary supplements such as prebiotics and probiotics, it could promote specific bacteria and help reshape the composition of our gut microbiome in the long term [140,144]. *Bifidobacterium*, *Lactobacillus* and *Streptococcus* are the common species used in probiotics [145], particularly the *Bifidobacterium* and *Lactobacillus* are the two most commonly used strains in human health studies [146,147]. Other potential probiotics such as Actinobacteria have also been shown to be useful for gut microbiome modulation in aquaculture or animal studies [148,149,150].

Probiotics are shown to improve general skin health. A study demonstrated the administration of *Lactobacillus paracasei* NCC2461 for two months to human subjects resulted in a drop in transepidermal water loss (TEWL) and skin sensitivity due to high transforming growth factor-beta (TGF-β), thus positively affecting the epidermal barrier integrity [34,151]. On top of that, the gut microbiome also impacts skin allostasis via both adaptive and innate immunity [103,142,152]. For instance, the administration of *L. paracasei* CNCM I-2116 (ST11) in a study demonstrated enhanced recovery of skin barrier function with less symptoms of reactive skin inflammation [153,154,155]. In an animal study where mice were administered with *Lactobacillus reuteri*, results showed that the mice treated with probiotic had rapid wound healing and shorter recovery time with high Foxp3+ regulatory T cells but no neutrophils [156].

There has been both animal studies and human studies demonstrating the effects of probiotics for improvement of psoriasis. In animal studies mainly involved imiquimod-induced psoriasis in mice, probiotics were generally found to improve psoriasis-like characteristics and suppressed proinflammatory cytokines IL-17 [142,157,158,159]. Some studies associated psoriasis with mediators of T cell activation, whereby probiotics help regulate T cells and decrease dryness and inflammation of the skin [160]. For instance, severe pustular psoriasis patients unresponsive to dapsone, methotrexate, and steroids displayed tremendous clinical improvement after receiving *Lactobacillus sporogenes* supplements 3 times a day for 2 weeks with nearly absolute remission after 4 weeks [161]. Study with psoriasis individuals fed with *B.infantis* 35624 for 8 weeks showed significant attenuation of TNF-α than those treated with placebo [143]. The alleviation of this inflammatory component possibly improves psoriasis symptoms. Other studies that involved human subjects also observed the effects of probiotics in improving quality of life, reducing psoriasis severity, preventing relapses, improving gut mineral absorption and downregulating proinflammatory markers [162,163,164,165]. Table 1 summarizes the current evidence on the use of probiotics in modulating skin homeostasis and treating psoriasis in both animal and human subjects. In general, *Lactobacillus* spp. improves skin homeostasis by reducing TEWL and strengthening skin barrier function. In subjects with psoriasis, *Lactobacillus* spp. and *Bifidobacterium* spp. reduces Th17 related cytokines and diminishes severity of psoriasis.

The use of probiotics to modulate the gut microbiome can also benefit the overall body’s immune modulatory function. Psoriasis and its comorbid diseases [166], are linked with high levels of circulating pro-inflammatory cytokines (IL-6, TNF-α). Probiotics have been shown to reduce the level of these cytokines and plasma C-reactive protein (CRP) [167,168]. A study on the immuno-regulatory effects of *B. infantis* on psoriasis subjects had showed a decrease in plasma levels of TNF-α and CRP, which indicated the effectiveness of *B. infantis* in reducing these pro-inflammatory biomarkers, and potentially treating psoriasis [143].

Probiotics have low risk of adverse effects as the majority of probiotics with bacteria producing lactic acid are nontoxigenic and nonpathogenic. On top of that there have been >70 clinical studies conducted on food with microbial ingredients with results showing probiotics has no adverse effects [100,169,170]. Studies have shown that the use of probiotics and prebiotics can improve cathepsin-L-like activity level (a measure of skin keratinocyte differentiation and indicator of skin barrier function) skin hydration and decrease the level of serum and urine phenol which are toxic by-products of the gut bacteria [171,172]. Thus, it is a good alternative treatment for improving and managing psoriasis and its comorbidities, as well as reducing side effects related to chronic use of other psoriatic medications. Along with the advances in transdermal drug delivery systems, future research could look into administration of active compounds of probiotics that helps with psoriatic lesions through the trans-epidermal route [173].

## 7. Discussion

Proteobacteria, Bacteroides, Actinobacteria, and Firmicutes makes up >98% of the gut microbiota and as mentioned earlier, the relative abundance of Proteobacteria and Bacteroides dropped while the relative abundance of Actinobacteria and Firmicutes rose in psoriasis patients which has been demonstrated by various studies [21]. The Firmicutes/Bacteroidota (previously known as Firmicutes/Bacteroidetes) (F/B) ratio is an important marker of the gut microbiota state [21], with several studies linking it with psoriasis comorbidities including obesity [174], nonalcoholic fatty liver disease [175], cardiovascular disease (CVD) [176], and insulin resistance [177]. For instance, high F/B ratio has been associated with CVD such as coronary artery disease [176]. The gut microbiome houses Proteobacteria and Firmicutes that convert dietary carnitine from eggs and red meat to trimethylamine (TMA), the precursor of pro-atherosclerotic metabolite trimethyl-amine-N-oxide (TMAO) which is a proatherogenic molecule independent of CVD risk factors [33,178,179]. TMAO changes cholesterol metabolism of the host and stimulates macrophage activation resulting in higher CVD risk, stroke, myocardial infarction, and death [141,178,180]. Besides that, it is proposed that TMAO is a candidate molecule for developing Type 2 diabetes mellitus. It was also found that high TMAO producers possess increased Firmicutes than Bacteroidota within stool samples [33].

The perturbation of F/B ratio can be seen in both psoriatic arthritis and psoriasis [114,118]. Based on a study, the F/B ratio is seen greatest in enterotype 2 (*Prevotella* is of predominance) psoriasis patients as compared to enterotype 1 (*Bacteroides* is of predominance) and enterotype 3 (*Ruminococcus* is of predominance) psoriasis patients [108]. After analyzing the differences within different enterotypes, it is hypothesized that a lower F/B ratio with type 2 enterotype has a higher risk of BT. Results showed that BT results in pro-inflammatory reaction and skin inflammation resulting in the need for aggressive treatment. This occurs probably due to gut microbial imbalance involving various bacterial groups, altering organic acid, and other compounds that lead to a pro-inflammatory state. However, neither was there any specific bacteria identified for BT when comparing healthy vs BT+/BT- psoriasis patients nor is there any significant difference when Psoriasis Area Severity Index (PASI) score of BT+ and BT- groups were compared. Overall, by acknowledging enterotype 2 psoriasis patients have higher risk of BT, it can be used as an indicator for detecting greater inflammatory responses and BT episodes in patient’s reaction to treatment [108].

Some subgroups under the phylum Bacteroidota have controversial roles such as *Bacteroides fragilis* and *Prevotella copri* [21]. *Bacteroides fragilis* impairs the intestinal barrier and contributes to inflammation by releasing enterotoxins whereas the non-toxigenic *Bacteroides fragilis* has advantageous anti-inflammatory characteristics via the production of polysaccharide A and SCFAs [181]. On the other hand, the relative abundance of *Prevotella copri* in psoriasis differs from other inflammatory diseases that shows a rise in relative abundance such as rheumatoid arthritis [182], and ankylosing spondylitis [183]. With conflicting data on the inflammatory and metabolic actions of *Prevotella copri*, there is inconclusive evidence on the beneficial and detrimental effect of *Prevotella copri* in the development of psoriasis [184]. It should be noted that *Prevotella copri* is made up of four clades rather than a monotypic species, accompanied by diet which can be another factor affecting the anti or pro-inflammatory state, thereby contributing to the conflicting data observed in many studies [185]. Dysbiosis of the gut microbiota composition is also modulated by the type of therapy and disease duration [182,183].

Under the phylum Firmicutes, studies have shown that families *Ruminococcaceae* and *Lachnospiraceae* increased in psoriasis compared with normal controls [21]. In terms of species, *Faecalibacterium prausnitzii* [124] and *Akkermansia muciniphila* [111,124] had decreased, while *Ruminococcus gnavus* was elevated in psoriasis [21]. About 5% of the gut bacteria belongs to the genus *Faecalibacterium* and its increase in level has been linked to the immune regulation [108,186]. *Faecalibacterium prausnitzii* is a beneficial microbe living in the large intestine producing SCFA butyrate which functions to decrease oxidative stress, supply energy for colonocytes and triggers Treg cells, allowing anti-inflammatory action hence conferring immune tolerance to sites other than the GI system [106]. The metabolites of *Faecalibacterium prausnitzii* can alter the pro-inflammatory response due to its protective effect on the gut barrier as well as the inhibition of NF-κB activation [187], thus other inflammatory diseases including ankylosing spondylitis and IBD also show a decreased in abundance of *Faecalibacterium prausnitzii* [124,183]. However, there are conflicting data where some studies showed the abundance of *Faecalibacterium prausnitzii* has no correlation with IBD, celiac disease etc. [188], whereas, others claim that the increase in *Faecalibacterium* spp. is related to inflammatory diseases including Crohn’s disease [189]. Nevertheless, *Akkermansia muciniphila* and *Faecalibacterium prausnitzii* affect psoriasis as these bacteria are responsible for SCFA production thus the anti-inflammatory actions [107,130]. Study found that decrease in *Akkermansia muciniphila* could possibly affect psoriasis progression and severity as it is inversely associated with cardiometabolic diseases, diabetes, obesity, and low grade inflammation [125]. However, the abundance of *Akkermansia muciniphila* could decrease the gut barrier function and body weight [190], while increase in *Ruminococcus gnavus* can lead to dysfunction of the gut barrier as it releases inflammatory polysaccharides and can be seen in IBD, coronary artery disease, eczema and spondyloarthritis [191].

The severity of psoriasis is also associated with the composition of the gut microbiome. Studies have shown a positive correlation between PASI and the blood concentration of intestinal barrier damage biomarkers. [192,193] Masallat et al., studied on fecal samples from 45 HC and 45 psoriasis subjects, reported that raised F/B ratio among psoriatic patients had a positive correlation with PASI score and greater abundance of Actinobacteria in HC was negative correlated with PASI [118]. However, some studies failed to establish the relationship between gut microbiome and severity of psoriasis. For instance, Chen et al., found no significant decrease in *F. prausinitizii* abundance in those with greater PASI score [112].

Despite the observed differences in taxa seen in psoriasis patients compared to healthy individuals, there is a lack of consistency of results involving the microbial diversity, directionality of differences or relative abundance. Besides that, studies using fecal samples to determine changes in gut microbiome may not be able to detect microbiome changes occurring in the intestine due to variation in composition of luminal- and mucosal-link microbiota [21,194]. It also does not provide information on whether the taxon identified is transient and nonviable or remains pathogenic. To study the gut microbiome better, the concurrent use of several sampling technique including colonic lavage, intestine bioptates, and mucosal–luminal interface aspirates may be applied besides the 16SrRNA gene profiling [21]. Nonetheless, unlike fecal sampling, these are invasive procedures and must be carried out by registered health professionals to avoid complications such as infection, perforation, and bleeding.

## 8. Conclusions

Dysbiosis of the cutaneous and intestinal microbiome play important roles in pathophysiology of psoriasis. Several clinical studies and research have shown the effects of gut microbiome on the pathogenesis of psoriasis, host homeostasis, allostasis and its association with distant organs beyond the GIT.

In terms of inflammatory skin diseases, the concept of gut–skin axis is a viable proposal for better skin condition. There is a difference in the composition of the gut microbiome of psoriasis patients when compared to healthy individuals. The gut and skin have their unique microbial community interacting with the immune system and the skin. Both inflammation of the gut and skin system involves IL-23/IL-17 signaling pathways. Thus, by regulating the gut microbiome, skin inflammation can be reduced via systemic immune system regulation.

Regardless, it is important to note that psoriasis is a heterogeneous disease, and the gut microbiome is dynamic which could vary due to gender, age, lifestyle, geographical background, diet, and medications. Alterations of gut microbiota could be affected by disease activity, comorbidities, disease duration, and treatment. All these could take time before it is seen which could possibly cause microbiome variations in both clinical and study participants, affecting the external validity. By further understanding the relationship between the gut microbiome and psoriasis pathogenesis, it is possible to develop novel prognosis and efficient treatment avenues. Modulation of the gut microbiome using probiotics could be a novel approach in preventing, managing, and even treating inflammatory skin diseases. Thus, maintaining a healthy gut microbiome is important to decrease intestinal permeability, decrease the risk of BT, and subsequently improve psoriasis.

## Figures and Tables

**Table 1:** Existing evidence of the relationship between probiotic supplementation and their effects on skin health and psoriasis.

Evidence of Benefits of Microbiome Modulation on General Skin Health				
Reference	Study Subjects	Probiotics Used	Duration of Intervention	Outcome of Treatment
[153]	Mice	*Lactobacillus helveticus*-fermented milk whey	5 weeks	Reduced TEWL and areas of dermatitis
[156]	Mice	*Lactobacillus reuteri* ATCC-PTA-6475	12 days	Shorter wound healing time, increased Foxp3+ regulatory T cell
[154]	Ex vivo human abdominal skin explant model	*Lactobacillus paracasei* CNCM-I 2116 (ST11)	24 h	Stronger skin barrier function, reduced neurogenic inflammatory skin diseases
[151]	Human	*Lactobacillus paracasei* NCC2461	Two months	Reduced transepidermal water loss (TEWL) and skin sensitivity
Evidence of benefits of microbiome modulation on psoriasis				
Non-human studies				
[142]	Mice	*Lactobacillus pentosus* GMNL-77	6 days	Improved skin lesions, reduced Th-17 associated proinflammatory cytokines, reduced Th17 and Th22 T cells in spleen
[157]	Imiquimod (IMQ)-induced psoriasis in mice	*Leuconostoc mesenteroides* NTM048	21 days	Reduced erythema and scaling, increased plasma deoxycholic acid (DCA) level, reduced IL-17 production in murine spleen
[158]	Imiquimod (IMQ)-induced psoriasis in mice	*Bifidobacterium adolescentis, Bifidobacterium breve, Bifidobacterium animalis, Lacticaseibacillus paracasei, Limosilactobacillus reuteri*	2 weeks	*B. adolescentis* CCFM667, *B. breve* CCFM1078, *Lacticaseibacillus paracasei* CCFM1074, and *Limosilactobacillus reuteri* CCFM1132 reduced psoriasis-like pathological characteristics and suppressed the release of IL-23/ Th17 related inflammatory cytokines
[159]	TNF-α-induced HaCaT cell hyperproliferation	102 strains of probiotics	Not specified	*Bifidobacterium animalis* CCFM1148 and *Lactobacillus paracasei* CCFM1147 suppressed keratinocyte hyperproliferation by preventing NF-κB activation and downregulating the levels of IL-6 and IL-8
Human studies				
[143]	Patients with psoriasis	*Bifidobacteria infantis* 35624	6–8 weeks	Reduced plasma C-Reactive Protein and TNF-α levels
[161]	Case study of a patient with pustular psoriasis	*Lactobacillus sporogenes*	6 months	Pustular lesions improved in 2 weeks and lesion free by 6 months of intervention
[162]	27 patients with psoriasis	*Bacteroides fragillis* BF839	12 weeks	Significant reduction in PASI score
[163]	50 patients with plaque psoriasis	Multistrain probiotic *including Lactobacillus acidophilus*, *Bifidobacterium* *bifidum*, *Bifidobacterium lactis* and *Bifidobacterium langum*	8 weeks	Reduced Beck’s Depression Inventory (BDI) and Dermatology Life Quality Index (DLQI) scores, reduced PASI and psoriasis symptom scale, increased Total Antioxidant Capacity (TAC), decreased CRP and IL-6 levels.
[164]	64 patients with mild to moderate psoriasis	Lactocare^®^ that contains seven strains (*Lactobacillus casei*, *Lactobacillus acidophilus*, *Lactobacillus rhamnosus*, *Lactobacillus bulgaricus*, *Bifidobacterium breve*, *Bifidobacterium longum*, *Streptococcus thermophiles* with prebiotic *fructooligosaccharide)*	12 weeks	Increased serum levels of Fe, Zn, P, Mg, Ca, and Na in probiotic group, signifying better mineral absorption
[165]	90 patients with plaque psoriasis	*Bifidobacterium longum* CECT 7347, *B. lactis* CECT 8145 and *Lactobacillus rhamnosus* CECT 8361	12 weeks	Greater reduction in PASI score, lower rate of relapse

## Data Availability

Data sharing not applicable.
